# The circadian activity rhythms for elderly inpatients with stroke or motor diseases in a rehabilitation facility and its relationship to physical activity level

**DOI:** 10.1007/s41105-023-00488-8

**Published:** 2023-09-29

**Authors:** Yuki Nakagawa, Kazue Noda, Yosuke Inoue

**Affiliations:** 1 Graduate School of Health Sciences, Kobe University, Kobe-shi, 654-0142 Japan; 2 Faculty of Health Sciences, Osaka University of Human Sciences, Settsu-shi, 566-8501 Japan; 3 Department of Rehabilitation, Kyowakai Medical Corporation Senri-chuo Hospital, Toyonaka-shi, 560-0082 Japan

**Keywords:** Rehabilitation, Physical activity, Circadian rhythm, Synthetic periodic regression, Patients

## Abstract

Disturbances in the circadian activity rhythms (CARs) of inpatients in rehabilitation facilities delay the recovery of physical and mental functions. The purpose of this study is to elucidate the circadian activity rhythms of hospitalized patients in a rehabilitation facilitie using the synthetic periodic regression analysis, and investigate the relationship between their physical activity levels and CARs.An observational study was conducted. A group of thirty-four inpatients participated in the study by wearing wrist-type activity monitors to measure metabolic equivalents (METs). Using synthetic periodic regression analysis, the CARs were analyzed based on the amount of physical activity throughout the day, and the exercise intensity classification of their physical activity was assessed. In the CARs of the inpatients, the mean physical activity level was 1.23 ± 0.09 METs. The maximum amount was 1.36 ± 0.15 METs. The range was 0.30 ± 0.15 METs. The maximum phase time was 11:48 ± 2:31 h. The longer the duration of physical activity over 1.6 METs, the higher the mean, maximum and range of the CARs. Physical activities with a METs level of 1.6 or higher might have an impact on the mean, maximum, and range of circadian activity rhythms in hospitalized patients.

**Supplementary Information:**

The online version contains supplementary material available at 10.1007/s41105-023-00488-8.

## Introduction

The circadian activity rhythms (CARs) of patients may be disrupted by hospitalization. This is because their lifestyle changes after admission, they experience stress, and may develop sleep disturbances [1, 2]. Especially in elderly patients, their CARs are more likely to be disrupted after hospitalization because their physical and mental functions have declined, making it difficult for them to adapt to the new lifestyle [3]. The disruption of the CAR delays the recovery for elderly inpatients with cerebrovascular, orthopedic, and other diseases who receive occupational therapy (OT) and physical therapy (PT) [4, 5]. Therefore, therapists need to understand the CARs of elderly inpatients.

There are several methods for assessing activity rhythm. These methods include conducting self-administered questionnaires [6], measuring electroencephalography [7], and measuring physical activity amount [8–10]. Among the various methods, using the activity logger for evaluating activity rhythm is recommended [11]. This method is less burdensome for patients and allows for continuous measurement of objective indices over a long period. In particular, the CAR evaluation using the 24-h or 12-h periodic regression analysis has proven to be effective [8–10].

The 24-h periodic CAR evaluation utilizes cosinor analysis [12–15], which quantifies and visually demonstrates CARs. However, it can sometimes mismatch the subject's original activity data [16], as it assumes that humans are only active with a 24-h or 12-h cycle per day [17–20]. In reality, people have multiple circasemidian cycles, including both a 24-h and a 12-h cycle [19, 21]. Therefore, analyzing CARs using both 24-h and 12-h cycles is necessary for accurately assessing inpatient CARs [22, 23]. Despite its importance, previous studies analyzing CARs for inpatients using both 24-h and 12-h periodic regression analysis are limited. It is crucial to comprehend inpatient CARs through 24-h and 12-h synthetic periodic regression analysis.

Furthermore, the CARs are analyzed based on the daily activity amounts of the patients, making it influenced by the activity itself. Clarifying the relationship between activity amounts and CARs can provide insights into the physical activity approaches of occupational therapists and physiotherapists, as well as suggestions for how hospitalized patients spend their time on the ward. Therefore, it was decided to investigate the relationship between CARs and activity levels.

The hypothesis is that activity levels are low, peak activity times are during the day in the CARs of hospitalized elderly patients and the CARs are related to physical activity amount [10, 24]. The purpose of this study is to elucidate the circadian activity rhythms of hospitalized patients in rehabilitation facilities using the synthetic periodic regression analysis, and investigate the relationship between their physical activity levels and CARs.

## Materials and methods

### Participants and recruitment

Participants were recruited from stroke or motor disease patients admitted to a rehabilitation facility between June 1, 2018, and May 30, 2021. These patients were transferred from an acute care hospital to the rehabilitation facility. In order to be included in the study, participants needed to be at least 65 years old and score 24 or higher on the mini-mental state examination (MMSE) [25]. Those with severe immobility, significant memory defects, aphasia, or consciousness disturbance were excluded from the study. Ultimately, 34 patients met the inclusion criteria and provided informed consent to participate. The study protocol was approved by the local ethical committee of Hospital A in Toyonaka, Japan (ethical numbers: 2018–06 and 2019–04). Data collection for each individual began one month after hospitalization to allow participants to become familiar with the hospital's daily schedule.

### The daily schedule and environment

The subjects spent their hospitalization following the hospital's schedule. At 7 a.m., the lights came on, and they proceeded to the dining room on the same floor of the ward to have breakfast by 7:30 a.m.. For those who were unable to move independently, hospital staff assisted them in moving to the dining room. After breakfast, they brushed their teeth and used the restroom before returning to their rooms. Between 9 a.m. and 12 p.m., the subjects received one rehabilitation session. During the time outside of rehabilitation sessions, they spent their time lying down or sitting on the bed in their rooms. At 12 p.m., they went to the dining room for lunch. After lunch, they brushed their teeth and used the restroom before returning to their rooms. From 1 p.m. to 5 p.m., two rehabilitation sessions were conducted. During the time outside of these sessions, they rested on their beds or sat in their rooms. At 6 p.m., they had dinner in the dining room, and after brushing their teeth and using the restroom, they returned to their rooms. They settled into bed by the lights-out time at 10 p.m.. Rehabilitation sessions, such as OT and PT, were conducted for 40 to 60 min per session. The subjects engaged in physical activities like stretching, strength training, and walking exercises, as well as activities of daily living (ADL) training. In their rooms, they had the freedom to engage in leisure activities, such as watching TV, listening to the radio, or reading around the bed area. However, for safety reasons, they were not allowed to freely go outdoors.

The illuminance in the hospital was measured using a digital lux meter (HOLDPEAK 881E, HOLDPEAK, Chaina). The illuminance in the hospital rooms was 300–350 Lux at eye level when lying in bed or sitting in bed during the day, and 1–5 Lux at eye level when lying in bed late at night. The illuminance in the platform or corridor of rehabilitation room during the day was 400–450 Lux at eye level when in a chair-sitting or standing position.

### Patients’ background characteristics

The data included as basic characteristics: the age, sex, body mass index (BMI), diagnosis, MMSE, presence of sleep medication, presence of psychotropic medication and the motor subtotal rating score of functional independence measure (motor FIM) were obtained from the medical records. The motor FIM consists of 13 items with a 7-point scale for independence [26]. The total motor FIM scores range from 13 to 91 points. The higher motor FIM scores indicate greater the level of ADL independence. The level of ADL independence refers to the degree of independence in activities such as eating, dressing, toileting, changing, bathing, and transferring. The participants were divided into two groups according to the locomotive faculty, either independent or dependent. The former could freely move around the wards by themselves, and the latter required assistance to move around.

### Amount of physical activity and sleep state

We used wrist actigraphs to measure sleep status and physical activity levels. We opted for wrist-type devices because waist-type activity monitors can sometimes come off when patients use the restroom, change clothes, or turn over in bed during sleep. Additionally, wearing two devices-one on the wrist and another on the waist-could be physically and mentally burdensome for elderly inpatients. Thus, to minimize discomfort, we chose to measure their physical activity solely with wrist-type actigraphs.

All participants were equipped with a wrist actigraph (Life Microscope, Hitachi, Tokyo, Japan) from Monday to Friday. Since weekends often included family visits that could disrupt participants' regular routines, we decided to collect actigraphy data continuously on weekdays and not on weekends. The actigraph was worn on the non-dominant hand. In cases where the non-dominant hand was paralyzed, the device was worn on the dominant hand instead. This wrist actigraph detects acceleration changes of 0.01 G/Rad/sec or more within the 2 to 3 Hz range. It calculates the amount of activity based on the acceleration change every 1 s and determines exercise intensity as metabolic equivalents (METs) [27, 28]. METs is a units of activity intensity. One METs indicates a resting state in the sitting position [29]. For example, easy work in a sitting position is about 1.5 METs, walking in the house is 2.0 METs [30].

Actigraphy was designed as a tool to monitor sleep conditions, and its accuracy has been confirmed through multiple studies [2, 31, 32]. This device used the Cole-Kripke algorithm to determine whether the person was in a state of sleep or wakefulness [33]. The sleep-related data included nocturnal sleep hours, sleep efficiency, waking time, and bedtime. To calculate sleep efficiency, the time spent in arousals during the sleep period was subtracted from the total nightly sleep time. This value was then divided by the total nightly sleep time. The data collected over five days were averaged to represent one day's worth of sleep information.

### Exercise intensity classification assessment of physical activity

The amount of physical activity was classified into three categories according to exercise intensity; 1.0 to 1.5 METs of activity was classified as sedentary behavior (SB), 1.6–2.9 METs as light-intensity physical activity (LIPA), and 3.0 METs and above were defined as Moderate-to-Vigorous Physical Activity (MVPA) [34]. The total SB, LIPA and MVPA hours per day were calculated from the averaged activity data. SB was calculated even after lights-off at night. Daytime SB hours were also calculated, except the hours from 22:00 h to 07:00 h the next morning.

### Synthetic periodic regression analysis

The 24-h and 12-h periods were examined for their appropriateness for this participants activity data, the therapists fitted 720 period components ranging from 24-h cycles to 3.3333E-8-h cycles to the subject's activity data. As a result, the 24-h and 12-h cycles fit the original data the best by the multiple contribution rate (R^2^). The multiple contribution rate, denoted as R^2^, is a statistical measure used to assess quantitatively how well the analyzed model fits the original data. It takes values between 0 and 1. A higher R^2^ value indicates that the regression model is better at capturing and explaining the patterns and the variation in the data.

A synthetic periodic regression analysis with 24-h and 12-h cycles was used [22, 23, 35]. The synthetic periodic regression curve is “ y = M + A1・cos (ω1・t–θ1) + A2・cos (ω2・t–θ2).” M (mesor) is the mean value of the synthetic periodic regression curve. A (amplitude) is the difference between the value from the mesor and the maximum or minimum value. A1 indicates the amplitude of the 24-h, and A2 indicates the amplitude of the 12-h periodic regression curve. θ (acrophase) is the phase angle of the maximum value in the periodic regression curve. θ1 indicates the acrophase of the 24-h, and θ2 indicates the acrophase of the 12-h periodic regression curve. The synthetic periodic regression analysis was conducted with advice from a statistics expert who has written many books on statistics in Japan.

### The CAR

The CAR was defined by six parameters. The mesor represented the average amount of activity in the day. The maximum value represented the highest level of activity. The maximum phase time represented the peak time of the day's activity [36]. The minimum value represented the lowest level of activity. The minimum phase time represented the calmest time of the day's activity [36]. The range represented the difference between the maximum and minimum values. A higher range value represented a greater balance of rest and activity in a day [36].

### Data analysis

The parameters of the CAR and sleep states were compared between the two groups: male and female, cerebrovascular and orthopedic disease, locomotive independent and dependent, and taking hypnotics and non-taking hypnotics. Welch’s test or the Mann–Whitney test was used. The relationship between the parameters of the CARs or sleep states and age, BMI, MMSE, motor FIM, total sleep time, sleep efficiency, waking time and sleeping time was analyzed with Pearson’s correlation or Spearman’s rank correlation.

The relationship between evaluation according to physical activity classification and the parameters of CAR as well as sleep states was analyzed with Spearman’s rank correlation coefficient. Significant correlations were found between SB, LIPA, and MVPA and the mesor, maximum, and range, respectively. For those parameters, single regression analysis with SB, LIPA, and MVPA was performed. Several confounding factors influencing Mesor, Maximum, and Range were examined using statistical methods such as intergroup comparisons and correlation. As a result, nocturnal sleep duration was found to be a confounding factor affecting the relationship between Mesor and either SB or LIPA. Therefore, we conducted multiple regression analyses considering the influence of nocturnal sleep duration in the relationship between Mesor and either SB or LIPA. Since Mesor was a time series data, the presence of autocorrelation using the Durbin-Watson test was verified. A Durbin-Watson statistic value close to 2 indicated no autocorrelation. The IBM SPSS Statistics 28.0.0.0 (IBM Corp., Armonk, N.Y., USA) was used to perform the statistical analyses. A p-value of less than 0.05 was considered evidence for statistical significance.

## Results

### CAR and evaluation according to physical activity classification

There were 13 men and 21 women. The mean ± SD age of the participants was 77.5 ± 7.7 yrs (range 66–90 yrs). Basic characteristics of 34 participants were listed in Table 1. The mean ± SD (range) of the CARs and sleep states of the participants were as follows. The mesor was 1.23 ± 0.09METs (1.09–1.50 METs). The maximum ± SD was 1.36 ± 0.15 METs (1.12–1.86 METs). The minimum ± SD was 1.05 ± 0.02 METs (1.02–1.10 METs). The range ± SD was 0.30 ± 0.15 METs (0.06–0.80 METs). The maximum phase time ± SD was 11:48 ± 2:31 h (7:44–18:09 h). The minimum phase time ± SD was 1:41 ± 0:40 h (0:36–3:05 h). R^2^ value was 0.72 ± 0.10 (0.49–0.87). Total sleep time was 458.0 ± 69.3 min. Sleep efficacy was 89.3 ± 6.4%. Waking time was 6.21 ± 0.89 h. Going to bed time was 21.84 ± 0.92 h.

**Table 1:** Basic characteristics of the participants

	All participants n = 34	Men n = 13	Women n = 21	p
Age (year)	77.5 ± 7.7	79.6 ± 6.3	76.1 ± 8.3	0.246^a^
Gender				
Male	13			
Female	21			
Body mass index (kg/m^2^)	22.3 ± 3.3	21.8 ± 3.9	22.6 ± 2.9	0.503^b^
Diagnosis				0.522^c^
Cerebrovascular syndrome	22	8	14	
Orthopedic disease	12	5	7	
Mini-mental state examination (score)	28.0 ± 1.7	28.3 ± 1.7	27.8 ± 1.7	0.381^a^
Hypnotics				0.525^c^
Yes	10	3	6	
No	24	10	15	
Psychotropic drugs				0.626^c^
Yes	2	1	1	
No	32	12	20	
Motor functional independence measure (score)	67.8 ± 17.4	64.2 ± 19.0	70.0 ± 16.4	0.400^a^
Locomotive independence				0.800^c^
Independence	20	8	12	
Dependence	14	5	9	

Data are mean ± standard deviation or n unless otherwise specified

^a^Mann-Whitney U test

^b^Two-sample t-test

^c^Chi-square test

The mean ± SD (range) of the evaluation according to the physical activity classification of the participants were as follows: The SB time was 22 h 7 min ± 2 h 19 min (15 h 21 min-24 h). The daytime SB time was 13 h 12 min ± 2 h 11 min (6 h 36 min-15 h). The LIPA was 1 h 53 min ± 2 h 17 min (0–8 h 27 min). The MVPA time was 0 min ± 2 min (0–12 min).

### The relationship between the CAR and baseline

**Fig. 1:**
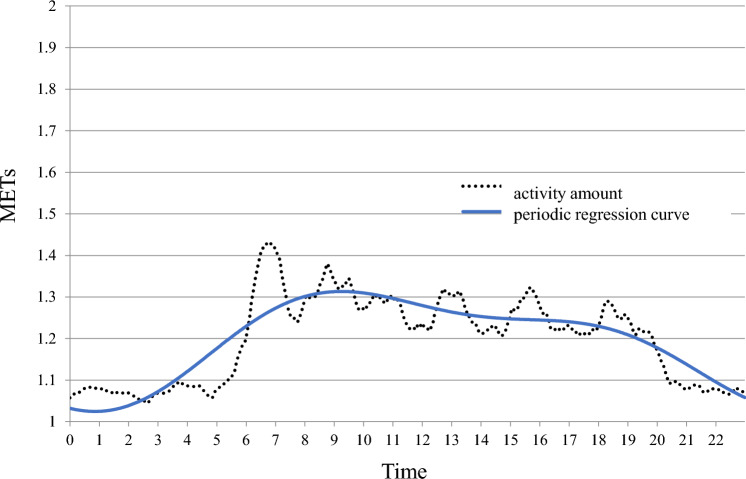
The maximum phase time is advanced (9:00 h). Man non-taking hypnotics. He wakes up around 5:00 h, is active around 9:00 h, and goes to bed around 20:00 h. He is an early bird

**Fig. 2:**
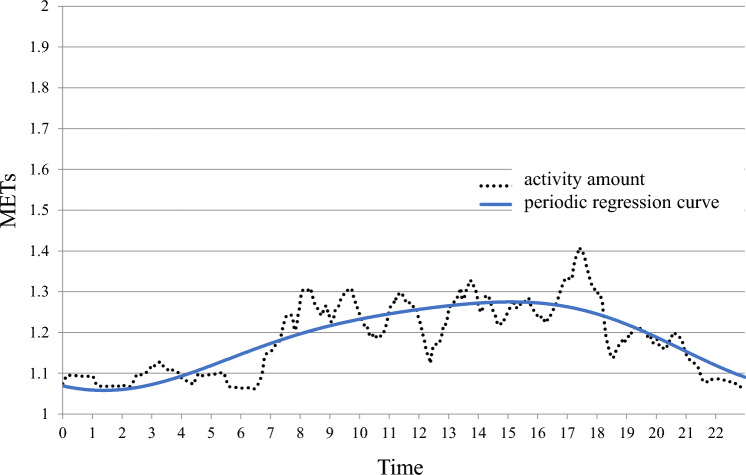
The maximum phase time is delayed phase (16:00 h). Woman taking hypnotics. She wakes up around 7:00 h, is active around 16:00 h, and goes to bed around 21:00 h. She is a night owl

Participants taking hypnotics had significantly slower maximum phase times than those not taking hypnotics (p = 0.022). Representative examples of CARs in hypnotics users and non-users were shown in Figs.1 and 2. Sleep status and other results were shown in Tables 2 and 3.

**Table 2:** Comparison of circadian activity rhythms and sleep patterns based on various basic information

Factors	Mesor (METs)	Maximum (METs)	Minimum (METs)	Range (METs)	Time max (h)	Time min (h)	Total sleep time (min)	Sleep efficiency (%)	Waking time (h)	Bed time(h)
Sex										
Male	1.20 ± 0.06	1.33 ± 0.11	1.06 ± 0.02	0.27 ± 0.12	10.98 ± 2.31	1.38 ± 0.46	479.8 ± 75.6	90.8 ± 4.3	6.02 ± 1.13	21.55 ± 0.97
Female	1.24 ± 0.10	1.39 ± 0.17	1.05 ± 0.02	0.33 ± 0.17	12.32 ± 2.56	1.88 ± 0.71	444.6 ± 63.3	88.4 ± 7.3	6.33 ± 0.71	22.01 ± 0.86
p	0.649^a^	0.675^a^	0.594^b^	0.484^a^	0.158^a^	0.030*^b^	0.153^b^	0.288^b^	0.333^b^	0.160^b^
Diagnosis										
CVA	1.23 ± 0.10	1.37 ± 0.16	1.06 ± 0.02	0.31 ± 0.16	12.17 ± 2.62	1.71 ± 0.63	460.8 ± 75.6	88.8 ± 7.1	6.13 ± 0.80	21.88 ± 1.01
Orthopedic disease	1.22 ± 0.08	1.36 ± 0.14	1.05 ± 0.03	0.31 ± 0.15	11.15 ± 2.27	1.66 ± 0.76	453.0 ± 58.8	90.2 ± 4.9	6.37 ± 1.05	21.76 ± 0.76
p	0.683^a^	0.763^a^	0.234^b^	0.79^a^	0.231^a^	0.842^b^	0.986^b^	0.543^a^	0.136^a^	0.724^b^
Locomotive										
Independence	1.24 ± 0.09	1.39 ± 0.16	1.06 ± 0.02	0.34 ± 0.16	11.84 ± 2.21	1.81 ± 0.74	452.3 ± 69.9	88.5 ± 6.3	6.30 ± 0.10	21.98 ± 0.89
Dependence	1.20 ± 0.08	1.32 ± 0.14	1.05 ± 0.02	0.27 ± 0.14	11.76 ± 2.98	1.52 ± 0.52	466.3 ± 70.3	90.5 ± 6.6	6.08 ± 0.73	21.63 ± 0.95
p	0.138^a^	0.231^a^	0.458^b^	0.231^a^	0.306^a^	0.058^b^	0.922^b^	0.609^b^	0.169^a^	0.281^b^
Hypnotics										
Yes	1.22 ± 0.07	1.37 ± 0.12	1.04 ± 0.02	0.32 ± 0.12	13.70 ± 2.99	1.83 ± 0.75	462.1 ± 60.5	91.2 ± 4.8	6.41 ± 0.71	22.04 ± 0.71
No	1.23 ± 0.10	1.36 ± 0.17	1.06 ± 0.02	0.30 ± 0.16	11.13 ± 1.98	1.64 ± 0.64	456.6 ± 73.4	88.6 ± 6.8	6.14 ± 0.95	21.76 ± 0.98
p	0.908^a^	0.759^a^	0.051^b^	0.591^a^	0.022*^a^	0.481^b^	0.842^b^	0.315^b^	0.432^b^	0.442^b^
Psychotropic drugs										
Yes	1.32 ± 0.08	1.53 ± 0.12	1.05 ± 0.03	0.48 ± 0.15	10.67 ± 1.40	1.30 ± 0.99	505.6 ± 26.9	90.1 ± 4.9	7.16 ± 0.25	21.72 ± 0.40
No	1.22 ± 0.09	1.35 ± 0.15	1.06 ± 0.02	0.30 ± 0.15	11.88 ± 2.57	1.72 ± 0.65	455.1 ± 70.3	89.3 ± 6.52	6.15 ± 0.88	21.84 ± 0.94
p	0.107^a^	0.071^a^	0.649^a^	0.071^a^	0.431^a^	0.513^a^	0.321^a^	1.000^a^	0.071^a^	0.602^a^

Data are mean ± standard deviation except for p value

*CVA* cerebrovascular accident, *locomotive* locomotive independence, *time max* the maximum phase time, *time min* the minimum phase time

^a^Mann-Whitney *U* test

^b^unpaired t-test

*p < 0.05

**Table 3:** Correlations between circadian activity rhythm and various factors

Factors	Mesor (METs)		Maximum (METs)		Minimum (METs)		Range (METs)		Time max (h)		Time min (h)		Total sleep time (min)		Sleep efficiency (%)		Wakingtime (h)		Bedtime (h)	
	r	p	r	p	r	p	r	p	r	p	r	p	r	p	r	p	r	p	r	p
Age (years)	– 0.162	0.36^a^	– 0.05	0.778^b^	– 0.090	0.619^a^	– 0.009	0.960^b^	– 0.112	0.528^b^	– 0.285	0.102^a^	– 0.12	0.499^a^	– 0.013	0.942^a^	0.014	0.936^b^	– 0.127	0.475^a^
Body mass index (kg/m^2^)	– 0.006	0.971^a^	– 0.058	0.745^b^	0.17	0.336^a^	– 0.097	0.586^b^	– 0.309	0.075^b^	– 0.24	0.171^a^	– 0.335	0.052^a^	– 0.123	0.488^a^	– 0.113	0.523^b^	0.088	0.620^a^
MMSE (score)	0.252	0.150^b^	0.191	0.280^b^	– 0.033	0.852^b^	0.150	0.398^b^	0.038	0.830^b^	0.117	0.509^b^	– 0.033	0.852^b^	0.008	0.964^b^	0.004	0.982^b^	0.225	0.201^b^
Motor FIM (score)	0.222	0.206^b^	0.196	0.266^b^	– 0.103	0.562^b^	0.208	0.237^b^	0.1616	0.363^b^	0.092	0.605^b^	– 0.25	0.154^b^	– 0.186	0.291^b^	0.133	0.452^b^	0.177	0.316^b^
Total sleep time (min)	– 0.488	0.003*^a^	– 0.317	0.068^b^	– 0.533	0.001*^a^	– 0.24	0.171^b^	0.176	0.320^b^	0.096	0.588^a^	***	***	0.687	0^a^	0.262	0.135^b^	– 0.454	0.007**^a^
Sleep efficiency (%)	– 0.622	0*^a^	– 0.306	0.078^b^	– 0.503	0.002*^a^	– 0.233	0.184^b^	– 0.025	0.887^b^	0.157	0.374^a^	0.687	0^a^	***	***	0.102	0.565^b^	– 0.189	0.285^a^
Waking time (h)	– 0.191	0.279^a^	– 0.173	0.329^b^	– 0.288	0.099	– 0.112	0.526^b^	0.309	0.076^b^	0.254	0.146^b^	0.262	0.135^b^	0.102	0.565^b^	***	***	0.256	0.144^b^
Bed time (h)	0.317	0.068^a^	0.186	0.292^b^	0.35	0.042*^a^	0.114	0.523^b^	0.378	0.028*^b^	0.270	0.123^a^	– 0.454	0.007*^a^	– 0.189	0.285^a^	0.256	0.144^b^	***	***
SB (min/day)	– 0.941	0*^b^	– 0.946	0*^b^	– 0.138	0.436^b^	– 0.922	0*^b^	– 0.076	0.668^b^	– 0.131	0.460^b^	0.306	0.079^b^	0.334	0.054^b^	0.129	0.468^b^	– 0.252	0.150^b^
LIPA (min/day)	0.941	0*^b^	0.946	0*^b^	0.136	0.443^b^	0.922	0*^b^	0.073	0.681^b^	0.13	0.462^b^	– 0.304	0.080^b^	– 0.333	0.054^b^	– 0.133	0.452^b^	0.248	0.158^b^
MVPA (min/day)	0.383	0.025*^b^	0.383	0.025*^b^	0.135	0.447^b^	0.371	0.031*^b^	0.019	0.913^b^	0.093	0.601^b^	– 0.369	0.032*^b^	– 0.396	0.021*^b^	– 0.147	0.408^b^	0.176	0.320^b^

Time max, the maximum phase time. Time min, the minimum phase time

*MMSE* mini mental state examination, *FIM* functional independence measure, *bed time* going to bed time, *SB* sedentary behaviour, *LIPA* light-intensity physical activity, *MVPA* moderate-to-vigorous physical activity

^a^Pearson correlation analysis

^b^Spearman correlation analysis

*p<0.05

### The relationship between the CAR and the evaluation according to physical activity classification

SB time was significantly associated with mean, maximum and range (p = 0.000, r = – 0.941, p = 0.000, r = – 0.946, p = 0.000, r = – 0.922, respectively). LIPA time was significantly associated with mean, maximum and range (p = 0.000, r = 0.941, p = 0.000, r = 0.946, p = 0.000, r = 0.922, respectively). The other results were shown in table 3.

### The single regression equations between the evaluation according to physical activity classification and the CAR

The results was shown the following: Mesor =—0.001 × SB(min/day) + 2.042 (F = 337.369, p = 0.001, R^2^ = 0.913), Mesor = -0.001 × SB(min/day) + 0.000 × Nocturnal sleep hours (min/day) + 2.072 (Multiple regression analysis: F = 194.158, p < 0.001, R2 = 0.926, Durbin-Watson statistic 2.056. Coefficient p-values: SB, p < 0.001; Nocturnal sleep hours, p = 0.028; VIF = 1.194), Mesor = 0.001 × LIPA(min/day) + 1.157 (F = 332.45, p = 0.001, R^2^ = 0.912), Mesor = 0.001 × LIPA (min/day) + 0.000 × Nocturnal sleep hours (min/day) + 1.234 (Multiple regression analysis: F = 191.620, p < 0.001, R2 = 0.925, Durbin-Watson statistic 2.061. Coefficient p-values: LIPA, p < 0.001; Nocturnal sleep hours, p = 0.027; VIF = 1.193), Mesor = 0.024 × MVPA(min/day) + 1.218 (F = 14.873, p = 0.001, R^2^ = 0.317).

Maximum =– 0.001 × SB(min/day) + 2.741 (F = 273.182, p = 0.001, R^2^ = 0.895), Maximum = 0.001 × LIPA(min/day) + 1.245 (F = 267.679, p = 0.001, R^2^ = 0.893), Maximum = 0.044 × MVPA(min/day) + 1.347 (F = 17.466, p = 0.001, R^2^ = 0.353).

**Fig. 3:**
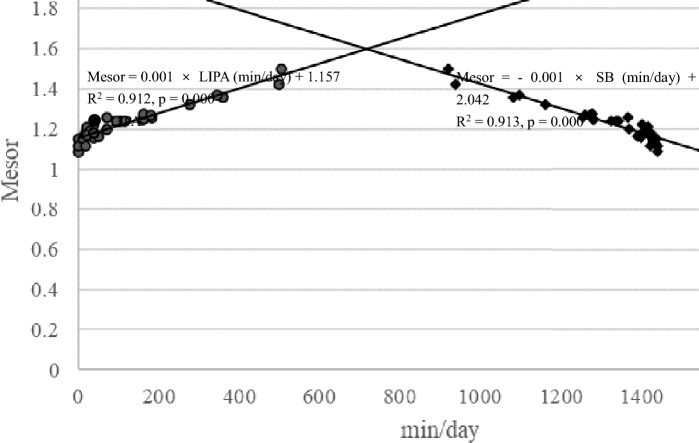
Simple regression analysis between mesor and SB or LIPA

**Fig. 4:**
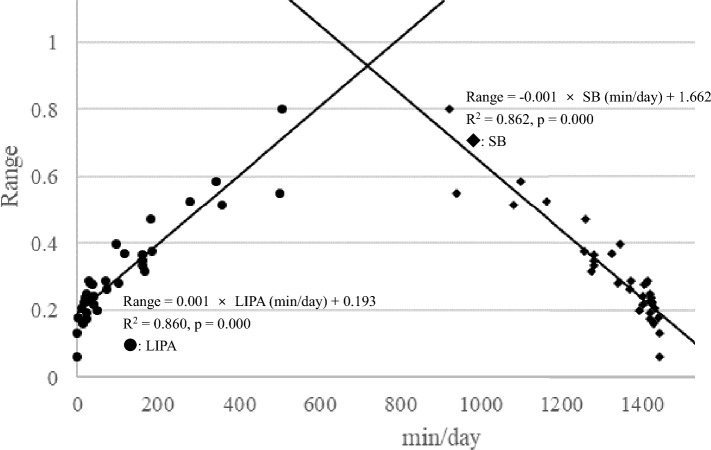
Simple regression analysis between range and SB or LIPA

Range =– 0.001 × SB(min/day) + 1.662 (F = 199.422, p = 0.001, R^2^ = 0.862), Range = 0.001 × LIPA(min/day) + 0.193 (F = 196.011, p = 0.001, R^2^ = 0.860), Range = 0.044 × MVPA(min/day) + 0.292 (F = 17.306 p = 0.001, R^2^ = 0.351). Simple regression analysis between mesor and SB or LIPA were shown in Fig. 3. Simple regression analysis between range and SB or LIPA were shown in Fig. 4.

## Discussion

### The characteristics of the CARs in participants

The participants engaged in sedentary behavior (SB) for approximately 22 h per day, with about 13 h of SB during the daytime. Light-intensity physical activity (LIPA) accounted for approximately 2 h, while moderate-to-vigorous physical activity (MVPA) was around 0.5 h. A previous study conducted in Japan reported SB time of 9 h, LIPA of 2.5 h, and MVPA of 0 h [37]. In comparison, the participants in our study (with FIM motor item scores of 68) exhibited lower levels of independence in ADL compared to those in the previous study (with FIM motor item scores of 82).

The average daily activity level of our subjects was approximately 1.23 METs, and the maximum activity level was about 1.36 METs. These values fall within the sedentary activity range (1.0 to 1.5 METs). The narrow range of daily activity, around 0.3 METs, suggests that the participants' physical and mental functions were impaired due to the disease, limiting their activity levels. Furthermore, the CAR reflects the overall pattern of physical activity throughout the day rather than simply capturing activity at one specific time [38].

The peak time (maximum phase time) of daily activity for our participants was observed between 9:15 h and 13:20 h, with an average around 12:00 h. To establish a reference, we considered previous studies that used single periodic regression analysis. In elderly inpatients, the peak time was around 13:30 h [14], whereas in community–dwelling elderly individuals, it ranged from 13:30 h to 16:00 h [36, 39, 40]. Our participants' peak time aligned with that of inpatients from the previous study, but it was about 3 to 4 h earlier than the community–dwelling elderly.

The difference in peak times between inpatients and community–dwelling individuals may be attributed to lifestyle variations. People living in the community may not necessarily wake up early in the morning, and some might perform housework in the evening or at night [41]. Additionally, they might be influenced by the nocturnal lifestyle of family members [42]. Conversely, our study's participants adhered to the hospital’s routine schedule and had limited evening or nighttime physical activity, leading to their peak activity time being in the morning.

### Relationship between basic characteristics and CAR

Regarding hypnotics, the peak time of daily activity for those taking these medications was about 2 h and 30 min later than those not taking them. However, there were no significant differences in waking time, bedtime, total sleep time, or sleep efficiency between the two groups. The participants who took hypnotics used medications like Rozerem, Brotizolam, and Zolpidem, which can cause drowsiness, fatigue, and lightheadedness as side effects [21]. If individuals taking hypnotics experience morning drowsiness due to these side effects, they might not be as active in the morning. Therefore, therapists should avoid scheduling OT or PT early in the morning for patients who are taking hypnotics. Regarding mobility and independence in daily activities, it was found that individuals with higher scores on the motor FIM or those who exhibit self-reliant movements are not necessarily showing higher mean, maximum, or range values in their CARs. It is speculated that this is attributed to the restricted environment of hospitalization, which limits the subjects' activity time and range, thus influencing the observed outcomes. In the case of the subjects, they were not allowed to freely move around inside or outside the hospital for safety reasons. Apart from scheduled activities such as occupational therapy, physical therapy, and meals, they spent their time lying in their hospital rooms. In spite of some subjects being self-reliant in mobility or having a high level of independence in their daily activities, their overall daily activity level was low due to spending a significant amount of time in their hospital rooms. The CARs reflect the overall activity pattern throughout the day rather than just the activity status at a particular moment. Therefore, it is considered that there were no significant differences in the parameters of CARs among the subjects.

### The relationship between the CAR and the amount of physical activity

The study found that the longer the sedentary behavior (SB) time, the lower the mesor, maximum, and range of the CARs. On the other hand, the longer the LIPA time, the higher the mesor, maximum, and range of the CARs. In simple terms, spending less time in a seated or lying position and more time engaging in activities with an intensity of 1.6 METs or higher led to higher values in the mean, maximum, and range of activity rhythms.

This is beneficial for physical function [36, 43], cognitive function [44], and the ability to perform activities of daily living (ADL) [45]. The World Health Organization (WHO) also emphasizes the importance of reducing sedentary behavior and increasing light-intensity physical activity to maintain good health and reduce the risk of cardiovascular disease and other illnesses [46]. Therefore, the study suggests that therapists should encourage physical activity and ward activities that involve an intensity of 1.6 METs or more to improve circadian activity rhythms and promote the overall health of the patients.

## Limitations

First, the subjects were admitted with a cerebrovascular or orthopedic disease. Therefore, their CAR may differ from those of patients admitted for other diseases. Second, this study was conducted at only one facility. It is not s generalized result. In the future, similar studies should be done at multiple facilities to determine the criteria for the patients’ CARs.

## Conclusion

The CARs in hospitalized elderly patients were investigated by synthetic periodic regression analysis. Physical activities with a METs level of 1.6 or higher might have an impact on the mean, maximum, and range of circadian activity rhythms in hospitalized patients.

### Supplementary Information

Below is the link to the electronic supplementary material.

Supplementary file1 (PDF 132 KB)

## Data Availability

There is no code availability.
