# Supplementary material for: The circadian activity rhythms for elderly inpatients with stroke or motor diseases in a rehabilitation facility and its relationship to physical activity level
Source: Sleep Biol Rhythms. 2023 Sep 29;22(1):125–35. doi: 10.1007/s41105-023-00488-8 (PMC10900012; doi:10.1007/s41105-023-00488-8)
Supplement: Supplementary file 1 — Supplementary file1 (PDF 132 KB) [file 41105_2023_488_MOESM1_ESM.pdf]

“The formulas for the synthetic periodic regression analysis with 24-hour and 12-hour cycles”

1． In periodic regression analysis, the following periodic regression model is assumed:

周期回帰モデル : $y = A_0 + \sum_{k=1}^{m} A_k \cos(\omega_k t - \theta_k) + \sum_{k=1}^{m} A_k \cos(\omega_k t - \theta_k)$

A_0 : 定数、メサー(MESOR)　A_k : 振幅(amplitude)　$\omega_k = \frac{360°}{T} k$: 角周波数(angular frequency)

t : 時間　T : 基本周期(fundamental period)　$\frac{T}{k}$: 周期成分kの周期(period)

θ_k : 位相(acrophase)　$\frac{\theta_k}{\omega_k}$: 位相時間　m≦(データ数)/2 : 周期成分数　ε : 回帰誤差

※周期回帰モデル：Periodic regression model、定数：constant term、時間：time、位相時間：phase time、周期成分数：number of periodic components、回帰誤差：regression error

2. The actual calculations

Regarding the assumed periodic regression model, it is calculated as a multiple regression model as follows.

$\beta_0 = \frac{a_0}{2}$　$\beta_1 = a_1$　$\beta_2 = b_1$　$\cdots$　$\beta_{2k-1} = a_k$　$\beta_{2k} = b_k$　$\cdots$　$\beta_{2m-1} = a_m$　$\beta_{2m} = b_m$

$x_0 = 1$

$$x_1 = \cos(\omega_1 t) = \cos\left(\frac{2\pi}{T} t\right) \qquad x_2 = \sin(\omega_1 t) = \sin\left(\frac{2\pi}{T} t\right)$$

:

$$x_{2k-1} = \cos(\omega_k t) = \cos\left(\frac{2\pi k}{T} t\right) \qquad x_{2k} = \sin(\omega_k t) = \sin\left(\frac{2\pi k}{T} t\right)$$

:

$$x_{2m-1} = \cos(\omega_m t) = \cos\left(\frac{2\pi m}{T} t\right) \qquad x_{2m} = \sin(\omega_m t) = \sin\left(\frac{2\pi m}{T} t\right)$$

$$\mathbf{y} = \mathbf{X}\boldsymbol{\beta} + \boldsymbol{\epsilon} = \hat{\mathbf{y}} + \boldsymbol{\epsilon}$$

$$\mathbf{y} = \begin{bmatrix} y_1 \\ \vdots \\ y_j \\ \vdots \\ y_n \end{bmatrix} \quad \mathbf{X} = \begin{bmatrix} 1 & x_{11} & \cdots & x_{1(2k-1)} & x_{1(2k)} & \cdots & x_{1(2m)} \\ \vdots & \vdots & \vdots & \vdots & \vdots & \vdots & \vdots \\ 1 & x_{j1} & \cdots & x_{j(2k-1)} & x_{j(2k)} & \cdots & x_{j(2m)} \\ \vdots & \vdots & \vdots & \vdots & \vdots & \vdots & \vdots \\ 1 & x_{n1} & \cdots & x_{n(2k-1)} & x_{n(2k)} & \cdots & x_{n(2m)} \end{bmatrix} \quad \boldsymbol{\beta} = \begin{bmatrix} \beta_0 \\ \beta_1 \\ \vdots \\ \beta_{2k-1} \\ \beta_{2k} \\ \vdots \\ \beta_{2m} \end{bmatrix} \quad \boldsymbol{\epsilon} = \begin{bmatrix} \epsilon_1 \\ \vdots \\ \epsilon_j \\ \vdots \\ \epsilon_n \end{bmatrix}$$

The least squares solution and various parameters are calculated using the least squares method as follows.

$$\hat{\beta} = [X'X]^{-1} X'y$$

$$A_0 = \frac{a_0}{2} = \hat{\beta}_0 \qquad a_k = \hat{\beta}_{2k-1} \qquad b_k = \hat{\beta}_{2k} \qquad A_k = \sqrt{a_k^2 + b_k^2} \qquad \theta_k^* = \tan^{-1}\left(\frac{b_k}{a_k}\right)$$
